# The effects of cannabidiol on nitric oxide synthases: a narrative review on therapeutic implications for inflammation and oxidative stress in health and disease

**DOI:** 10.1186/s42238-025-00332-5

**Published:** 2025-09-29

**Authors:** Seyed Amin Alavi Hooshmand, Maryam Rameshrad, Amirhossein Sahebkar, Mehrdad Iranshahi

**Affiliations:** 1 Biotechnology Research center, Pharmaceutical Technology Institute, Mashhad University of Medical Sciences, Mashhad, Iran; 2 Department of Pharmacodynamics and Toxicology, School of Pharmacy, Mashhad University of Medical Sciences, Mashhad, Iran; 3 Pharmaceutical Research Center, Mashhad University of Medical Sciences, Mashhad, Iran; 4 Center for Global Health Research, Saveetha Medical College and Hospitals, Saveetha Institute of Medical and Technical Sciences, Saveetha University, Chennai, India; 5 Biotechnology Research Center, Pharmaceutical Technology Institute, School of Pharmacy, Mashhad University of Medical Sciences, Mashhad, Iran; 6 Applied Biomedical Research Center, Basic Sciences Research Institute, Mashhad University of Medical Sciences, Mashhad, Iran

**Keywords:** Cannabidiol, Nitric oxide synthase, Nitric oxide, Inflammation, Oxidative stress, Cardiovascular health, Neuroprotection, Cancer

## Abstract

Cannabidiol (CBD), a non-psychoactive compound from *Cannabis sativa*, shows promise as a therapeutic agent for conditions associated with inflammation and oxidative stress, often involving nitric oxide (NO) signaling dysregulation. This review summarizes preclinical and clinical data on CBD’s impact on nitric oxide synthase (NOS) isoforms and NO levels in cardiovascular, neurological, metabolic, and immune systems. Studies suggest that CBD can reduce inflammation-induced inducible NOS (iNOS) expression while maintaining or enhancing endothelial NOS (eNOS)-mediated NO production, leading to decreased oxidative stress, improved endothelial function, and reduced neuroinflammation. The effects of CBD vary based on dose, formulation, timing, and disease state, with potential interactions with metabolites and other drugs affecting safety. Further research is needed to determine optimal dosing, formulation, pharmacokinetics, metabolite profiles, and long-term safety for specific conditions.

## Graphical abstract

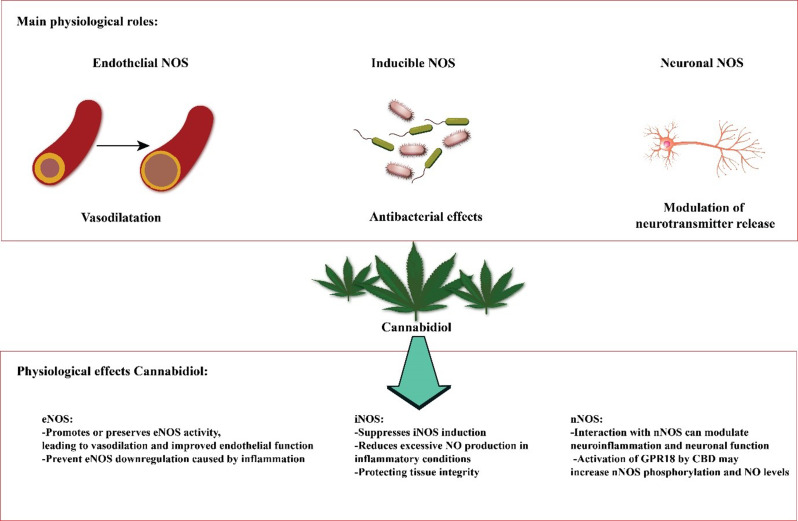

## Introduction

One of the most prominent phytochemicals in *C. sativa* is cannabidiol (CBD), which is recognized for its non-intoxicating nature and favorable safety profile. CBD was approved by the U.S. Food and Drug Administration (FDA) under the brand name Epidiolex^®^ in 2018 for treating severe drug-resistant epilepsy in children, and it has received international recognition for its efficacy in managing spasticity related to multiple sclerosis (MS) (Table 1) (Zhou et al. 2023). Additionally, it holds orphan drug designation for potential use in treating neonatal hypoxic-ischemic encephalopathy. An array of preclinical and clinical studies have demonstrated that CBD possesses a variety of beneficial properties, including antioxidant, immunomodulatory, anti-inflammatory, neuroprotective, antipsychotic, procognitive, anti-anxiety, and anti-proliferative effects (Castillo et al. 2010; Gorelick et al. 2022; Jeong et al. 2019; Baranowska-Kuczko et al. 2021; Kicman and Toczek 2020). These attributes render CBD a candidate for addressing numerous clinical conditions such as cardiovascular diseases (Kicman and Toczek 2020), hypertension (Baranowska-Kuczko et al. 2021), cancer (Jeong et al. 2019), metabolic disorders (Gorelick et al. 2022), and neurodegenerative diseases (Castillo et al. 2010).

**Table 1:**
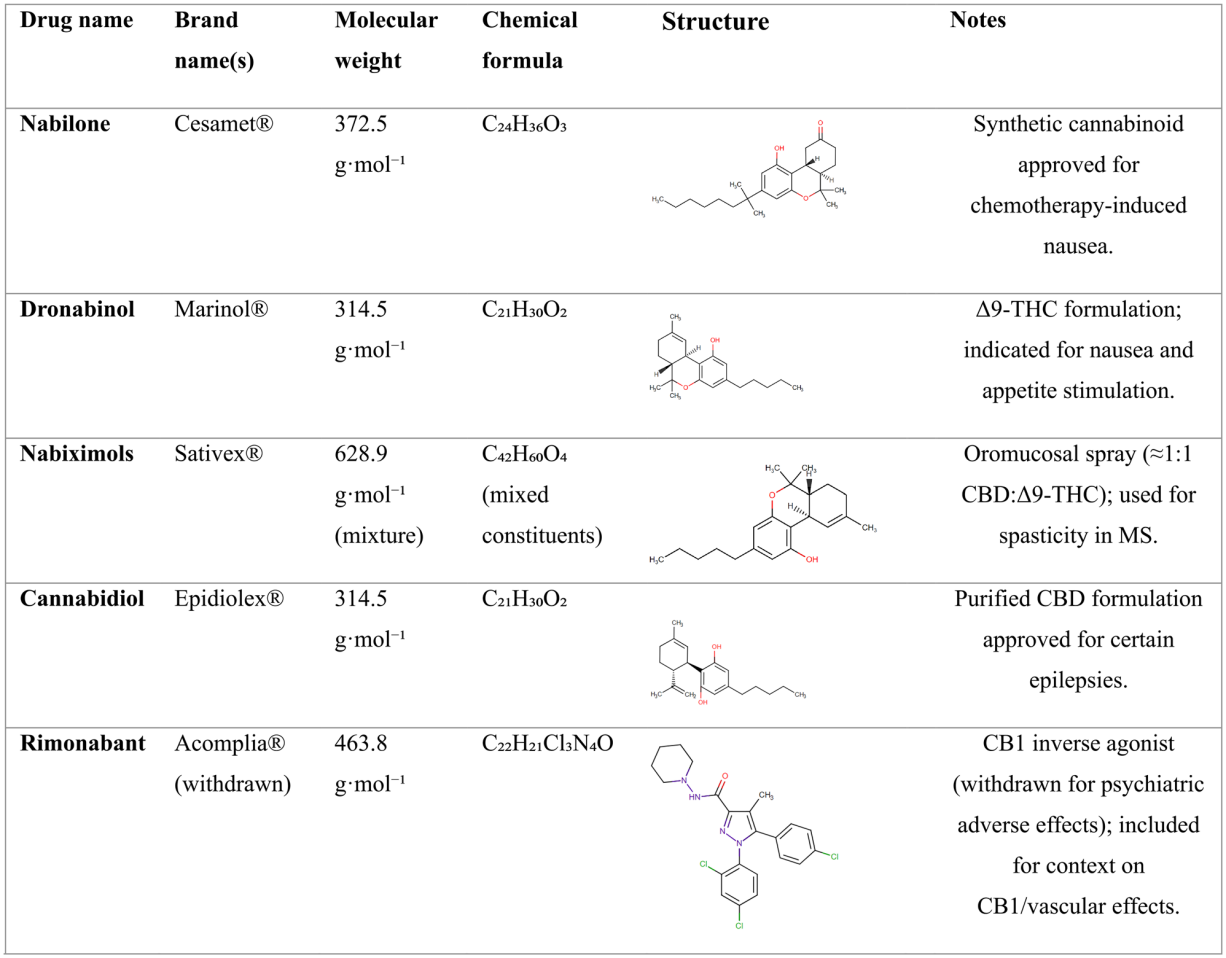
Pharmacological properties of selected cannabinoids (FDA-approved / notable**)**

Inflammation and oxidative stress are central, interrelated processes in many acute and chronic disease states (Socco et al. 2017; Zamanian et al. 2023). There is extensive evidence regarding the anti-inflammatory properties of various phytochemicals (Asgary et al. 2013; Giosia et al. 2022; Iranshahi et al. 2009; Saberi-Karimian et al. 2020; Gurgenci et al. 2023). A key molecular mediator at the intersection of these processes is nitric oxide (NO), a vital signaling molecule whose dysregulation contributes significantly to disease pathogenesis. NO, a vital signaling molecule involved in various physiological processes, is synthesized through a reaction catalyzed by nitric oxide synthase (NOS). There are three distinct isoforms of NOS, each with specific sites of expression and functions (Król and Kepinska 2021). Recent studies have increasingly highlighted the role of individual NOS isoforms and their dysregulation in the pathogenesis of several diseases, including metabolic disorders, cancer, and cardiovascular conditions. Notably, a deficiency in eNOS-derived NO is a primary contributor to endothelial dysfunction, while excessive iNOS-driven NO and nitrosative stress can cause cellular damage. Such dysregulation is linked to a range of diseases, including atherosclerosis, hypotension, diabetes, hypercholesterolemia, and neurodegenerative disorders (Król and Kepinska 2021; Kashfi 2020; Pozo et al. 2024; Erhabor et al. 2024). Given its broad anti-inflammatory and antioxidant profile, a critical area of research involves understanding CBD’s interactions with the NOS/NO system, a nexus of redox and inflammatory signaling.

Emerging evidence suggests that CBD can modulate NOS expression and activity, decreasing pro-inflammatory iNOS while potentially preserving or enhancing protective eNOS function (Chen et al. 2023). This nuanced modulation could underpin many of CBD’s therapeutic benefits. However, the effects appear to be highly context-dependent, varying by tissue type, disease state, and dosage.

This narrative review aims to fill this gap by critically synthesizing and evaluating current preclinical and clinical evidence on how CBD affects specific NOS isoforms and NO signaling. The central research question is: How does CBD’s modulation of NOS isoforms contribute to its therapeutic effects against inflammation and oxidative stress in various diseases? We will explore this question across cardiovascular, neurological, immune, metabolic, and oncological systems to provide a comprehensive understanding of the mechanism. Additionally, we will discuss the impact of CBD formulations, safety considerations, and emerging areas like epigenetic regulation to offer insights into the therapeutic potential of targeting the NOS pathway with CBD.

## Methodology

A literature search was conducted using databases such as PubMed, Scopus, and Web of Science. The search included keywords such as “Cannabidiol,” “Cannabinoids,” “CBD,” “phytocannabinoid,” “Epidiolex,” “cannabidiolum,” “cannabinol,” “cannabinodiol,” and “eNOS,” “Endothelial NOS,” “Endothelial Nitric oxide synthase,” “iNOS,” “inducible NOS,” “inducible Nitric oxide synthase,” “nNOS,” and “neuronal NOS,” “Neuronal Nitric oxide synthase.” The literature search was limited to studies published in the last 20 years until August 2025. Studies that investigated the effects of CBD on NOS pathways in various conditions and disorders, including cardiovascular, neurological, metabolic disorders, cancers, and immune diseases, were included regardless of article type, such as reviews and original articles, with a priority given to clinical trials, in vitro studies, in vivo studies, and meta-analyses. On the other hand, studies that did not focus on CBD’s interaction with NOS, those lacking peer-reviewed status, and duplicate reports were excluded.

### Different type of cannabinoid and their signaling pathways

**Fig. 1:**
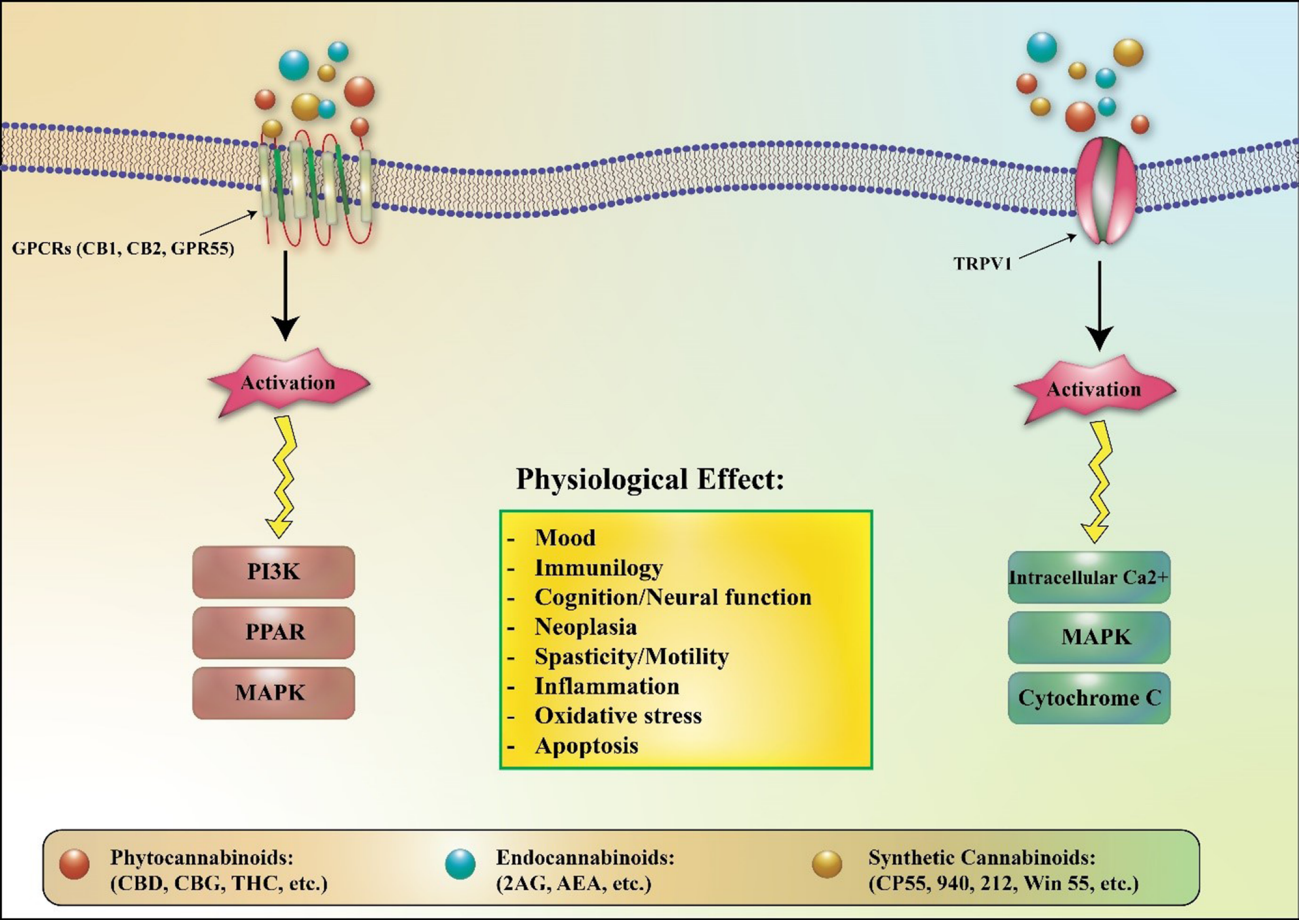
Cannabinoid signaling pathways and their effects. Cannabinoids, whether derived from plants (phytocannabinoids), produced internally (endocannabinoids), or synthesized artificially (synthetic cannabinoids), primarily engage with G-protein-coupled receptors (GPCRs) like the CB1 and CB2 receptors, as well as GPR55. Additionally, they may activate transient receptor potential channels such as TRPV1. The resulting signaling pathways differ according to the specific receptor that is activated, leading to various physiological effects on pain perception, appetite regulation, mood alterations, and numerous other bodily functions

It is crucial to understand that numerous cannabinoids, including Δ9-THC, influence pharmacological and physiological processes by interacting with the endogenous cannabinoid system. In contrast, certain cannabinoids, such as CBD, exhibit a low affinity for these receptors and operate outside the traditional endocannabinoid framework. Anandamide, recognized as the first identified endocannabinoid and referred to as N-arachidonoylethanolamine (AEA), is produced from membrane phospholipid precursors (Devane et al. 1992; Opitz et al. 2007; Maccarrone 2017). At elevated nanomolar concentrations, anandamide functions as a partial agonist at both CB1 and CB2 cannabinoid receptors, acts as a potent partial agonist at the G protein-coupled receptor 55 (GPR55), and demonstrates low-affinity full agonist activity at the transient receptor potential vanilloid 1 (TRPV1) calcium channel. Moreover, various TRP channels and receptors from the PPAR, adrenoceptor, and serotonin families can also interact with cannabinoid ligands. The most prevalent endocannabinoid, 2-arachidonoylglycerol (2-AG), is synthesized from phosphatidylinositol 4,5-bisphosphate that contains arachidonic acid. Notably, 2-AG demonstrates greater efficacy at CB1, CB2, and GPR55 receptors compared to AEA. In addition to endocannabinoids, cannabinoids are also derived from two other sources: phytocannabinoids, which are found in Cannabis plants, and synthetic cannabinoids, which are artificially created in laboratories and can engage cannabinoid receptors (Fig. 1) (Bisogno et al. 1999; Hanlon et al. 2015; Murataeva et al. 2014).

### Mechanisms and different type of NOS

**Fig. 2:**
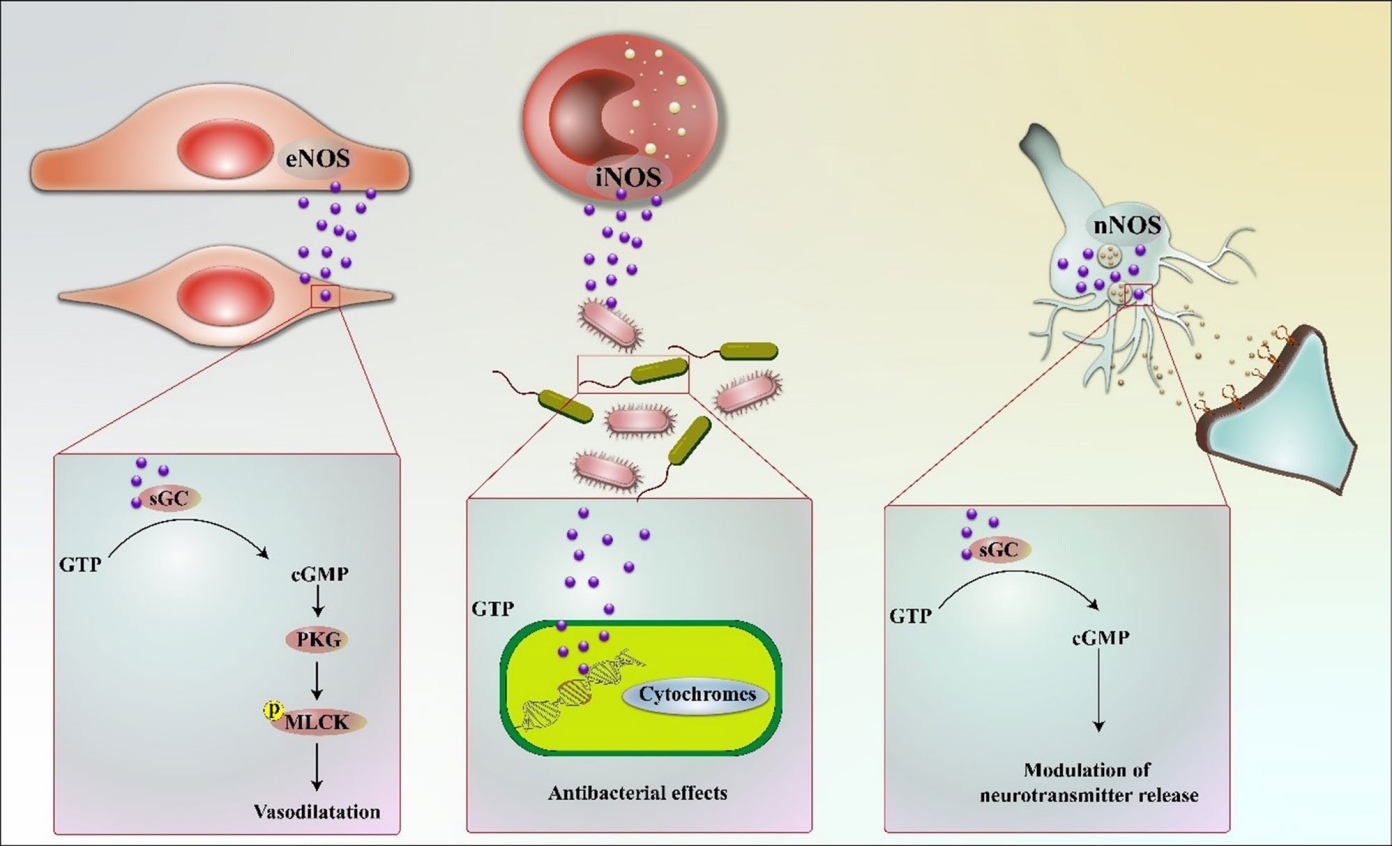
Signaling mechanisms of the three nitric oxide synthases. This activation results in the production of cyclic guanosine monophosphate (cGMP), which activates protein kinase G (PKG) and subsequently phosphorylates myosin light chain kinase (MLCK), culminating in smooth muscle relaxation. nNOS is primarily present in neurons at both sides of the synaptic cleft, where it plays a critical role in modulating neurotransmitter release through cGMP-dependent pathways. iNOS is expressed in various cells, including immune and epithelial cells, in response to proinflammatory cytokines or bacterial components. Once activated, iNOS produces significant amounts of NO over extended periods, which at elevated concentrations can exert cytotoxic effects on pathogens through multiple mechanisms, including the inhibition of essential enzymes involved in replication and cellular respiration (Pourbagher-Shahri et al. 2021)

NO in mammals is produced by three discrete isoforms of NOS, each encoded by separate genes: inducible NOS (iNOS), neuronal NOS (nNOS), and endothelial NOS (eNOS) (Fig. 2). All NOS proteins function as homodimers (Rosselli et al. 1998). eNOS is predominantly localized in the endothelial cells that line the interior of blood vessels. Upon stimulation, eNOS produces NO, which diffuses to adjacent smooth muscle cells, leading to the activation of soluble guanylate cyclase (sGC). Additionally, NO can be generated through NOS-independent mechanisms, specifically via the sequential reduction of nitrate (NO_3_^−^) and nitrite (NO_2_^−^). Under low partial pressure of oxygen (pO_2_), NO_2_^−^ can be reduced to NO by metal-containing enzymes such as deoxymyoglobin, xanthine oxidase (XO), and deoxyhemoglobin (deoxyHb/deoxyMb). These NOS-independent pathways for NO production are referred to as the NO_3_^−^/NO_2_^−^/NO pathway or O_2_-independent NO formation (Ledo et al. 2021). The significance of NO signaling, along with the functions of NO donors and NOS inhibitors, is essential for comprehending the underlying mechanisms of age-related diseases and exploring potential therapeutic approaches (Pourbagher-Shahri et al. 2021).

### Signaling mechanisms of endocannabinoid in crosstalk with NO levels

The endocannabinoid system (ECS), comprising cannabinoid receptors CB₁ and CB₂, endogenous ligands (anandamide and 2-AG), and metabolic enzymes (FAAH, MAGL) (Zou and Kumar 2018), exhibits complex bidirectional interactions with NO pathways. These interactions significantly influence neuroinflammation, neuronal function, and peripheral homeostasis.

CB₁ receptor activation demonstrates dual effects on NO synthesis. In the CNS, it suppresses neurotoxic iNOS expression in microglia and astrocytes by inhibiting Nuclear Factor kappa-light-chain-enhancer of activated B cells (NFκB) and glutamate excitotoxicity (Cabral et al. 2001; Esposito et al. 2006), conferring protection against β-amyloid toxicity and hypoxic-ischemic injury (Snow and Albensi 2016; Fernández-López et al. 2006). Conversely, CB₁ enhances nNOS activity via Gi/o-mediated extracellular-signal-regulated kinase (ERK)/c-Jun N-terminal kinases (JNK) pathways (Maroso et al. 2016; Kim et al. 2006), potentially impairing cognition despite promoting neurogenesis. In peripheral tissues, CB₁ upregulation elevates iNOS while suppressing eNOS activity(Rutkowska and Fereniec-Gołębiewska 2009; Tyryshkin et al. 2010), contributing to mitochondrial dysfunction in hepatocytes and vascular dysregulation.

Activation of CB2 receptors reduces inflammatory signaling by decreasing iNOS expression in central immune cells such as microglia and astrocytes (Gómez-Gálvez et al. 2016; Oddi et al. 2012). This is achieved by inhibiting the NF-κB pathway through CB2 receptor coupling to the cAMP/PKA signaling cascade in microglial cells (Tao et al. 2016). Additionally, CB2 receptor activation suppresses iNOS in cardiomyocytes, leading to a reduction in ischemia-reperfusion injury (González et al. 2011; Schmidt and Walter 1994).

The differential regulation of NOS isoforms by ECS signaling cascades reveals distinct mechanistic pathways. Both nNOS and eNOS isoforms are activated through CB₁ and CB₂ receptor-mediated stimulation of cAMP/PKA, PI3K, and PKC signaling pathways (Yen et al. 2011; Hurt et al. 2012), with their activity being further dependent on Ca²⁺/calmodulin complex formation (Förstermann et al. 1994; Förstermann and Sessa 2012). In contrast, the iNOS is suppressed by cannabinoid receptor activation through reduction of NFκB nuclear translocation (Snow and Albensi 2016; Fernández-López et al. 2006).

Notably, NO reciprocally modulates ECS function via S-nitrosylation of GPCRs (Kayki-Mutlu and Koch 2021). This post-translational modification alters β-arrestin recruitment and clathrin-mediated endocytosis (Morris et al. 2022). Chronic hypernitrosylation disrupts Gi/o and Gαs coupling ((Morris et al. 2022; Jin et al. 2018), impairing PI3K/Akt and ERK signaling (Mahavadi et al. 2014).

These intricate EC-NO interactions highlight therapeutic opportunities for neurodegenerative and cardiovascular disorders through receptor-specific modulation of NOS activity (Park et al. 2016; Saha and Pahan 2006). In the Tables 2 and 3, we summarized the various effects of CBD based on NOS pathways involvement across different experimental models.

**Table 2:** Selected experimental studies on CBD (and related cannabinoids) with NOS/NO-relevant outcomes

Ref	Model (in vitro / in vivo)	Dose / regimen (as reported)	Key NOS/NO-related outcome(s)	Mechanistic link to NOS
Stanley et al. 2015	Human aortic endothelial cells (in vitro)	10 µmol·L⁻¹, 10 min	↑ phospho-eNOS (Ser1177); Akt/ERK phosphorylation; endothelial-dependent vasorelaxation ex vivo	CBD activates PI3K/Akt→eNOS signaling, increasing NO production and vasodilation.
Baranowska-Kuczko et al. 2021	DOCA-salt and SHR rats (in vivo)	10 mg·kg⁻¹ daily ×2 weeks	Improved endothelium-dependent vasodilation; effects blocked by L-NAME	Functional evidence that CBD enhances NO-mediated vasorelaxation (NOS-dependent).
Matouk et al. 2018	Diabetic rats; GPR18 activation (in vivo)	abn-CBD, regimen as reported	↑ eNOS expression, ↑ NO and cGMP; improved cardiac function	Cannabinoid/GPR18 activation restores eNOS/NO signaling → cardioprotection.
Rajesh et al. 2007	Human coronary artery endothelial cells (in vitro, high-glucose)	4 µM ×48 h	↓ mitochondrial superoxide, ↓ NF-κB activation, ↓ iNOS, ↓ ICAM-1/VCAM-1	CBD prevents HG-induced iNOS upregulation and preserves endothelial integrity.
Rodrigues et al. 2024	BV2 microglia (in vitro)	1 µM ×1 h (LPS model)	Inhibition of NLRP3 inflammasome and ↓ iNOS, ↓ NO, ↓ IL-1β/TNF-α	PPARγ/NLRP3 pathway links CBD to reduced microglial iNOS/NO production.
Tanikawa et al. 2023	Neuropathic pain model (mouse, in vivo)	20 mg·kg⁻¹ i.p. ×21 days	Antinociception mediated by NO and S-nitrosylation of KATP channels	Shows a physiological role for NO in peripheral CBD effects.
Jeong et al. 2019	CRC cell lines (in vitro, oxaliplatin resistance)	4 µM ×12 h ± oxaliplatin	CBD suppressed eNOS phosphorylation and NO; induced ROS-mediated autophagy → restored chemosensitivity	CBD targets eNOS/NO to overcome NO-dependent chemoresistance.
Shi et al. 2023	Melanoma (B16-F10) in vitro / in vivo	2.5 µg·mL⁻¹ (cells) / 5 mg·kg⁻¹ i.p. ×7 d (mice)	↓ tumor growth; ↓ serum iNOS and inflammatory cytokines	Antitumor and anti-inflammatory effects associated with lowered iNOS/NO.
Callejas et al. 2018	Gastroschisis rat model (in vivo)	30 mg·kg⁻¹ daily ×3 days	↓ nitrite/nitrate; ↓ tissue iNOS expression	Direct reduction of tissue nitrosative markers and iNOS.
Fouad et al. 2012	Doxorubicin cardiotoxicity (rat, in vivo)	5 mg·kg⁻¹ daily i.p. ×4 weeks	↓ cardiac NO and iNOS; ↓ oxidative/nitrative stress; improved cardiac markers	Chemoprotective effect via suppression of iNOS/NO and oxidative damage.

**Table 3:** Clinical / ex-vivo human evidence related to CBD and NO signaling

Ref	Type of evidence	Key finding(s)	Relevance to NOS/NO
Stanley et al. 2015	Human arterial tissue (ex-vivo)	CBD induced endothelium-dependent vasorelaxation (requires intact endothelium)	Supports eNOS/NO involvement in CBD-mediated vasodilation in human vessels.
—	Clinical trials / human RCTs	**Limited clinical trial data directly assessing CBD effects on NOS/NO in patients.**	Indicates a gap - mechanistic preclinical evidence is strong, but translation to humans needs targeted trials.

### The effects of cannabidiol on NOS in cardiovascular disorders

NO is a critical signaling molecule in various organ systems, particularly within the cardiovascular system, where it plays multiple vital roles. While it was initially recognized primarily as a vasodilator promoting the relaxation of smooth muscle and thereby facilitating increased blood flow NO is now understood to influence numerous other cardiovascular functions, including endothelial and myocardial enhancement, as well as neuronal signaling (Roy et al. 2023). Importantly, NO is synthesized through the classical L-arginine–NO synthase pathway, as well as via the recently identified enterosalivary nitrate-nitrite-NO pathway, which underscores the significance of dietary nitrate found in fruits and vegetables. This novel pathway suggests that dietary nitrate may confer cardioprotective effects, supported by clinical trials demonstrating that increased nitrate intake can improve blood pressure, enhance endothelial function, mitigate ischemia-reperfusion injury, reduce arterial stiffness, optimize platelet function, and boost exercise performance, all while showing an increase in markers indicating improved NO status. Overall, these findings illuminate the multifaceted role of NO in cardiovascular health and its potential as a target for dietary and therapeutic interventions (Bondonno et al. 2016).

Numerous studies have demonstrated that CBD exerts protective effects against cardiovascular disorders by modulating NOS activity and influencing NO levels, thereby impacting cardiac health and function (Naya et al. 2024). This supports CBD’s early clinical safety profile in a vulnerable cohort. In this section, we reviewed the most important of this research. In one previous in vivo study by Amr A Fouad et al. highlighted the protective effects of CBD against renal ischemia/reperfusion injury in rats, showing that CBD administration (5 mg/kg, intravenous) significantly mitigated oxidative stress markers and histopathological damage associated with renal ischemia (Fouad et al. 2012). Specifically, CBD reduced NO levels that were elevated due to ischemia/reperfusion, while also decreasing the expression of iNOS, a key mediator of inflammation and tissue injury during oxidative stress. This suggests that CBD can attenuate the detrimental effects of ischemia on NOS activity, promoting renal recovery (Fouad et al. 2012). Another in vivo study investigating the cardioprotective effects of CBD against doxorubicin-induced cardiac injury demonstrated that daily CBD treatment (5 mg/kg, intraperitoneal) not only decreased serum markers of cardiac damage, such as creatine kinase-MB and troponin T, but also significantly reduced levels of cardiac malondialdehyde, tumor necrosis factor-alpha (TNF-α), NO, and calcium ions (Fouad et al. 2013). In a mouse heart failure model induced by Ang II and L-NAME, subcutaneous CBD reduced cardiac fibrosis and hypertrophy, preserved ejection fraction and mitochondrial function, and maintained redox balance. Although NOS wasn’t the focus, preserved mitochondrial and contractile function suggests secondary stabilization of physiological NO pathways (García-Rivas et al. 2025). Additionally, CBD treatment increased reduced glutathione levels, which are crucial for antioxidant defense. The immunohistochemical analysis showed a notable reduction in the expression of iNOS and other pro-inflammatory mediators in cardiac tissue, supporting the notion that CBD helps to modulate NOS activity and protect cardiac integrity (Fouad et al. 2013). Further clinical/in vitro research by Stanley et al. has shed light on the mechanisms underlying the vascular effects of CBD, revealing its ability to induce endothelium-dependent vasorelaxation in human mesenteric arteries. This vasorelaxant effect is mediated through the activation of CB1 and transient receptor potential channels (Stanley et al. 2015). Importantly, CBD treatment led to increased phosphorylation of eNOS, indicating enhanced NO production. The interplay between CBD, NO, and NOS suggests that CBD may serve as a vasodilatory agent, providing beneficial effects on vascular health, particularly in conditions characterized by endothelial dysfunction (Stanley et al. 2015). In a recent study in 2024, Tepebaşı et al. examining the effects of CBD on LPS-induced cardiovascular damage, it was found that CBD treatment significantly improved pathological and biochemical markers of inflammation. The treatment reduced oxidative stress and preserved eNOS expression, which was downregulated in the LPS group. By modulating intracellular signaling pathways involving interleukin-6 (IL-6), Hif1α, and STAT3, CBD effectively countered systemic inflammation and enhanced NO signaling, thereby providing cardiovascular protection (Tepebaşı et al. 2024).

#### Mechanistic insights

CBD appears to have a contextual effect on NO biology in cardiovascular disorders. It suppresses the pathological induction of iNOS and excessive NO/nitrosative stress in cases of inflammatory or ischemic injury. In models of endothelial dysfunction, CBD promotes or preserves physiological eNOS activity by mechanisms such as phosphorylation through Akt/ERK pathways and reducing inflammatory mediators that impair eNOS function. The anti-inflammatory and antioxidant properties of CBD, including inhibition of NF-κB and decreased proinflammatory cytokines, provide a mechanistic explanation for these actions. This suggests that CBD limits iNOS induction during inflammation and supports eNOS-dependent vasoprotection when the endothelium is intact or undergoing repair.

Overall, current evidence indicates that CBD modulates NOS/NO in a context-dependent manner, suppressing harmful iNOS-driven NO in inflammatory or ischemic conditions while enhancing protective eNOS signaling in endothelial and vascular models. These effects are mediated through anti-inflammatory, antioxidant, and kinase-mediated signaling pathways.

### The effect of cannabidiol on NOS in neurological disorders

Cannabidiol is increasingly recognized for its neuroprotective properties in various neurological disorders. Notably, one of its crucial mechanisms of action is the modulation of NOS, particularly eNOS and iNOS. This part integrates findings from several studies that evaluate the effects of CBD on NOS dynamics within the context of neuroprotection and inflammation in neurological disorders.

### Alzheimer’s disease

CBD has been shown to have beneficial effects in Alzheimer’s models by reducing iNOS expression and NO release through NF-κB inhibition. It also enhances Nrf2 antioxidant pathways and modulates cytokines such as IL-10 (Hickey et al. 2024). Esposito et al. discovered that CBD inhibited iNOS and pro-inflammatory cytokines, such as IL-1β, in an AD mouse model, leading to a reduction in hippocampal neuroinflammation (Esposito et al. 2007). An in-silico study identified eNOS as a primary target of CBD, affecting cerebral blood flow and inflammation. Modified CBD analogs exhibited enhanced binding to proteins associated with AD, indicating a potential for improved therapeutic efficacy (Choi et al. 2023). In vivo model of tauopathy and neurodegeneration, Sativex^®^ (1:1 CBD:Δ9-THC) reduced iNOS-driven neuroinflammation and oxidative stress independently of CB1/CB2 receptors, underscoring CBD’s receptor-agnostic antioxidant properties (Casarejos et al. 2013).

#### Mechanistic insight

CBD reduces Alzheimer’s-related neuroinflammation primarily by inhibiting NF-κB-driven iNOS expression and NO release while activating Nrf2 antioxidant responses and shifting cytokine balance (e.g., ↑IL-10, ↓IL-1β), thereby lowering oxidative stress and hippocampal inflammation (Hickey et al. 2024; Esposito et al. 2007). Complementary actions include modulation of cerebral blood flow via eNOS-related targets (García-Rivas et al. 2025), receptor-agnostic antioxidant effects when combined with THC (Sativex^®^) in tauopathy models, and the potential for improved efficacy with CBD analogs that show enhanced binding to AD-related proteins (Choi et al. 2023; Casarejos et al. 2013).

### Huntington’s disease

Cannabidiol and related cannabinoids protect the brain from oxidative damage by exerting antioxidant effects and providing neuroprotection through microglial CB₂ receptor activation (Tomaszewska-Zaremba et al. 2025). These processes are crucial for reducing oxidative stress associated with Huntington’s disease. Sagredo et al. investigated a 1:1 combination of CBD and Δ9-THC (similar to Sativex) in HD models. This treatment decreased iNOS, oxidative stress, and neuroinflammation without activating CB1/CB2 receptors, highlighting CBD’s antioxidant and anti-inflammatory properties in HD progression (Sagredo et al. 2011). Research by Rodrigues et al. has shown that CBD inhibits the NLRP3 inflammasome and iNOS in in BV2 microglial cells, reducing NO, IL-1β, and TNF-α through PPARγ activation, independent of CB2 receptors (Rodrigues et al. 2024).

#### Mechanistic insight

CBD and related cannabinoids provide neuroprotection by reducing oxidative stress and neuroinflammation through various NOS- and inflammasome-related pathways. CBD decreases iNOS expression and NO production, mitigates oxidative stress, and inhibits pro-inflammatory mediators (IL-1β, TNF-α) through NF-κB and PPARγ signaling, without the need for CB1/CB2 receptor activation (Sagredo et al. 2011; Rodrigues et al. 2024). Combination therapies like CBD:Δ9-THC (1:1) can amplify antioxidant and anti-inflammatory properties, demonstrating their potential in delaying HD progression.

### Depressive disorders

Wang et al. demonstrated that CBD reduced the expression of pro-inflammatory markers, including iNOS, TNF-α, and IL-1β mRNA, in the cortex and hippocampus of depressed mice. This reduction in neuroinflammation was associated with observed improvements in anxiety and cognition (Wang et al. 2021a, b). Furthermore, in an in vivo study, Spencer et al. found that inhibiting nNOS, in combination with N-acetylcysteine (NAC), reduced THC/CBD-seeking behavior in rats, suggesting a link between nNOS and addiction mechanisms (Spencer et al. 2018). Recent preclinical studies link CBD’s antidepressant-like effects to its anti-inflammatory actions. In vivo, Poudel et al. (2024) showed that CBD treatment reversed chemotherapeutic stress-induced depressive behaviors and noted that CBD ‘inhibits iNOS expression via PPARγ (Poudel et al. 2024). CBD’s PPARγ-mediated inhibition of iNOS and NLRP3 in microglia implies it could reduce neuroinflammatory NO signaling in depression These studies confirm that CBD has antidepressant effects through various mechanisms, including reducing neuroinflammation driven by iNOS, enhancing anti-inflammatory signaling mediated by PPARγ, and providing neuroprotective effects that alleviate depressive behaviors.

#### Mechanistic insight

CBD downregulates iNOS, TNF-α, and IL-1β in cortical and hippocampal regions, improving anxiety and cognition (Choi et al. 2023), while PPARγ-mediated inhibition of iNOS and NLRP3 further reduces microglial-driven inflammation (Poudel et al. 2024). Additional evidence suggests a role for nNOS in addiction-related behaviors, as nNOS inhibition reduced THC/CBD-seeking when combined with NAC (Spencer et al. 2018). Collectively, CBD’s antidepressant-like effects arise from iNOS suppression, PPARγ-dependent anti-inflammatory pathways, and neuroprotective modulation of NO signaling.

### Neuroprotective effects

CBD demonstrates strong neuroprotective effects by modulating NOS isoforms, particularly iNOS and nNOS, in various neurological disorders. Recent in vivo research on models of intraventricular hemorrhage (IVH), CBD reduces brain damage by suppressing iNOS-mediated oxidative stress and preserving blood-brain barrier integrity, thereby alleviating long-term cognitive deficits (Pozo et al. 2024). Similarly, in cerebral ischemia, cannabinoids like dexanabinol protect against neuronal death by reducing NO levels, highlighting nNOS as a potential therapeutic target (Durmaz et al. 2008). In a more recent in vitro investigation, CBD protected rat neural cell cultures from oxidative stress-induced damage (e.g., via reduced ROS and improved cell viability) but not excitotoxicity, highlighting its selective antioxidant neuroprotective role without direct NOS modulation in this context (Jantas et al. 2024).

Moreover, in vitro research by Kim et al. shows that CBD counteracts NO’s inhibition of neurogenesis through CB1 receptor activation, while an NOS inhibitor (7-nitroindazole) enhances this process, confirming NO’s role in limiting brain repair In an in vivo autoimmune encephalomyelitis (EAE) model, which mimics multiple sclerosis, CBD was shown to reduce levels of iNOS and nitrotyrosine, indicating a reduction in oxidative stress and neuroinflammation (Giacoppo et al. 2015). Study by Castillo et al. on hypoxic-ischemic injury (in vivo) demonstrate that CBD downregulates iNOS through CB2/adenosine receptors, reducing apoptosis and oxidative damage (Castelli et al. 2014). In the context of methamphetamine (METH) neurotoxicity, overactivation of nNOS leads to peroxynitrite formation and neuronal damage; Δ9-THC, structurally similar to CBD, reduces nNOS levels, suggesting that cannabinoids may mitigate nNOS-related excitotoxicity (Castelli et al. 2014).

CBD demonstrates strong neuroprotective effects by modulating NOS isoforms, particularly iNOS and nNOS, in various neurological disorders, including through suppression of oxidative stress and preservation of neuronal integrity across both in vivo and in vitro models.

### Suppression of neuroinflammation

CBD effectively suppresses neuroinflammation by targeting iNOS and associated pathways. In vitro studies have shown that combining CBD with cannabigerol (CBG) inhibits iNOS, modulates NF-κB signaling, and increases anti-inflammatory cytokines (IL-10, IL-37), while synergistically activating the Nrf2 antioxidant pathway (Mammana et al. 2019).

CBD-dihydroartemisinin conjugates (e.g., C3D) have been found to suppress LPS-induced NO production in microglia by inhibiting iNOS and IL-1β through NF-κB blockade, demonstrating superior efficacy compared to CBD alone in the recent in vitro investigation (Wang et al. 2021a, b). Moreover, in vivo research conducted by Alonso et al. in 2023 on Dravet syndrome, CBD reduces astroglial/microglial activation and iNOS-linked inflammation in the prefrontal cortex and hippocampus, improving behavioral deficits (Alonso et al. 2023). Lebanese cannabis oil (59.1% CBD) inhibits LPS-induced iNOS and COX-2 in monocytes by suppressing MAPK pathways (ERK/JNK/p38), confirming the multi-target anti-inflammatory action of CBD (Shebaby et al. 2021).

Fleisher-Berkovich et al. have highlighted that CBD-rich cannabis cultivars consistently inhibit LPS-induced iNOS/NO in glial cells, underscoring the cultivar-specific efficacy in controlling neuroinflammation (Fleisher-Berkovich et al. 2024).

CBD effectively suppresses neuroinflammation by targeting iNOS and associated pathways, such as NF-κB and NLRP3, leading to reduced NO production, cytokine modulation, and enhanced antioxidant responses. Recent evidence reinforces its multi-receptor mechanisms in microglial models.

### Potential in treating neuropathic pain

CBD shows significant potential in treating neuropathic pain by selectively modulating NOS isoforms. Research indicates that nNOS plays a crucial role in CBD-induced analgesia. A key study found that peripheral pain relief by CBD and other analgesics, such as morphine, was eliminated by the nNOS inhibitor L-NPA in hyperalgesic rats, confirming nNOS-derived NO as an essential mediator. Inhibitors of eNOS or iNOS did not affect this outcome, emphasizing the specificity of nNOS (Romero et al. 2011).

Additionally, a recent in vivo study by Almeida et al. demonstrated that the dose-dependent pain relief from systemic CBD in neuropathic pain involves the PI3Kγ/nNOS/NO signaling pathway, as selective nNOS inhibition reversed the analgesic effects. Importantly, inhibiting iNOS did not impact the effects of CBD, highlighting nNOS as the primary therapeutic target (Almeida et al. 2024). Costa et al. identified eNOS as a key target of CBD in sciatic nerve injury models. Oral administration of CBD (2.5–20 mg/kg) reduced thermal and mechanical hyperalgesia by inhibiting NO production through eNOS without significantly suppressing iNOS. This selectivity suggests that CBD avoids disrupting NOS broadly, potentially reducing excitatory neurological side effects (Costa et al. 2007).

The anti-inflammatory synergy of CBD was demonstrated in combination therapies. When combined with Morinda citrifolia extract (MCS-ext), CBD enhanced NO suppression in LPS-stimulated macrophages compared to CBD alone (in vitro), likely through NF-κB inhibition downstream of iNOS. This combination therapy preserved cell viability, indicating a safer therapeutic profile (Tanikawa et al. 2023).

CBD has shown promise in treating neuropathic pain by selectively modulating NOS isoforms, particularly nNOS and eNOS, through pathways like PI3Kγ/NO/KATP. This allows for targeted analgesia with minimal impact on other isoforms. Additionally, CBD’s anti-inflammatory properties can be enhanced in combination therapies, such as with CBG or conjugates, to target iNOS and NF-κB pathways, suppressing NO and cytokines. This offers improved efficacy and safety for neuroinflammatory conditions.

### CBD regulation of NOS in cancer: chemoprotection and antitumor effects

INOS plays a crucial role in the link between inflammation and cancer, as chronic overexpression of iNOS can lead to DNA damage and carcinogenesis through sustained NO production (Rao 2004).

CBD has been shown to counteract this process by suppressing iNOS-mediated oxidative and nitrosative stress. For example, in cases of cisplatin-induced nephrotoxicity, an in vivo study by Pan et al., CBD has been found to reduce renal iNOS expression, peroxynitrite formation, and cell death, thereby preserving kidney function (Pan et al. 2009). Similarly, another in vivo study conducted by Hao et al., CBD has been shown to attenuate doxorubicin cardiotoxicity by reducing iNOS-driven oxidative stress and improving mitochondrial function (Hao et al. 2015). A recent study by Tabatabaei et al. (2024) in a 4T1 breast cancer mouse model found that combining CBD with doxorubicin reduced cardiac iNOS levels, decreased matrix metalloproteinases (MMP2/MMP9), and increased antioxidant SOD2. This combination helped alleviate chemotherapy-induced inflammation and oxidative stress without affecting the treatment’s effectiveness against tumors (Tabatabaei et al. 2024). CBD’s protective effects also extend to reducing NF-κB/NOX4 in arsenic-induced kidney damage (an in vivo) (Vadizadeh et al. 2024).

In various cancers such as colorectal cancer (CRC) and prostate cancer, both in vitro and in vivo studies have shown that CBD triggers apoptosis by increasing reactive oxygen species (ROS) levels, leading to mitochondrial damage, endoplasmic reticulum (ER) stress, and activation of caspases. This process does not directly involve NOS but emphasizes the role of oxidative signaling as a crucial mechanism for CBD’s anti-tumor effects (Mashabela and Kappo 2024). Furthermore, Pongking et al. (2024) demonstrated in vitro and in vivo in gemcitabine-resistant cholangiocarcinoma (a biliary cancer model) that CBD induced ROS production (measured by DCFH-DA), ER stress (upregulating CHOP and ATF4), and apoptosis, significantly inhibiting tumor growth in xenografts (Pongking et al. 2024).

CBD has been demonstrated to decrease aberrant crypt foci and tumor incidence, partly through antioxidant pathways that protect DNA and reduce proliferation. While CBD inhibited phospho-Akt, it did not alter iNOS or COX-2 overexpression in this context (Aviello et al. 2012). Moreover, In non-cancer contexts, CBD has been shown to protect nucleus pulposus cells from H₂O₂-induced damage by reducing iNOS, COX-2, and pro-inflammatory cytokines (IL-1β, IL-6) while enhancing anti-apoptotic Bcl-2 (Aviello et al. 2012). Preclinical studies in a colon cancer model showed that CBD treatment reduced NO production while enhancing apoptosis (Liu 2025). Endothelial NOS also plays a role in cancer progression, particularly in oxaliplatin-resistant CRC cells where increased phosphorylated eNOS (NOS3) leads to elevated NO production, promoting cell survival and chemoresistance. The combination of CBD with oxaliplatin has been shown to suppress eNOS phosphorylation and NO levels, triggering ROS-mediated autophagy and restoring tumor sensitivity to chemotherapy (Jeong et al. 2019). Moreover, CBD has demonstrated anti-angiogenic effects in breast cancer cells (MCF-7) by reducing NO levels and enhancing antioxidant activity (glutathione, catalase) (Erhabor et al. 2024).

Novel delivery systems, such as effervescent CBD microneedles (Ef/CBD-SD@DMNs), have been developed to improve CBD efficacy. These microneedles have been shown to reduce serum iNOS and pro-inflammatory markers in melanoma *in vitro/in vivo* models, enhancing transdermal CBD delivery and activating tumor-suppressive TRPV1 pathways. While the specific effects on NOS were secondary, the overall outcome was a reduction in inflammation (Shi et al. 2023).

#### Mechanistic insights

CBD reduces oxidative and nitrosative stress in tumors and protects organs by decreasing iNOS expression and NO/peroxynitrite formation (Pan et al. 2009; Hao et al. 2015; Tabatabaei et al. 2024; Vadizadeh et al. 2024). It induces apoptosis in various cancers, including CRC, prostate cancer, and cholangiocarcinoma, through ER stress and caspase activation (Mashabela and Kappo 2024; Pongking et al. 2024). In oxaliplatin-resistant CRC, it promotes autophagy and resensitizes tumors to chemotherapy by suppressing eNOS and increasing ROS (Jeong et al. 2019). CBD impairs angiogenesis in breast cancer cells by reducing NO bioavailability and enhancing antioxidant defenses (Erhabor et al. 2024). Novel delivery systems like effervescent CBD microneedles enhance transdermal delivery, suppress inflammatory mediators, and activate tumor-suppressive pathways (Shi et al. 2023).

CBD has a dual-action role in cancer models. It offers chemoprotection by suppressing iNOS/eNOS-driven NO and peroxynitrite formation, reducing inflammation, and providing antitumor effects by promoting ROS accumulation, ER stress, apoptosis, autophagy, and anti-angiogenesis. These effects can restore chemosensitivity. Enhanced delivery systems, such as effervescent microneedles, can enhance these benefits. Overall, preclinical data suggest that CBD could be a valuable addition to cancer therapy and should be further investigated in clinical trials.

### Effect of cannabidiol on NOS in the immune system

CBD exhibits a wide range of immunomodulatory effects, primarily through its interaction with various receptors and pathways involved in inflammatory processes. One of its significant mechanisms of action is the inhibition of iNOS expression, contributing to the attenuation of inflammation. Elevated iNOS levels are commonly found in various conditions characterized by chronic inflammation, such as autoimmune diseases, neurodegenerative disorders, and tissue injury which in this section are defined (Giacoppo et al. 2015; Zorrilla et al. 2024; Rajan et al. 2016; Li et al. 2022; Frodella et al. 2024). New data on autoimmune inflammation support CBD’s role in downregulating iNOS. In a RA model, a high-CBD extract significantly reduced joint inflammation, building on prior reports that CBD lowers iNOS expression in synovial cells (Aswad et al. 2025).

**Fig. 3:**
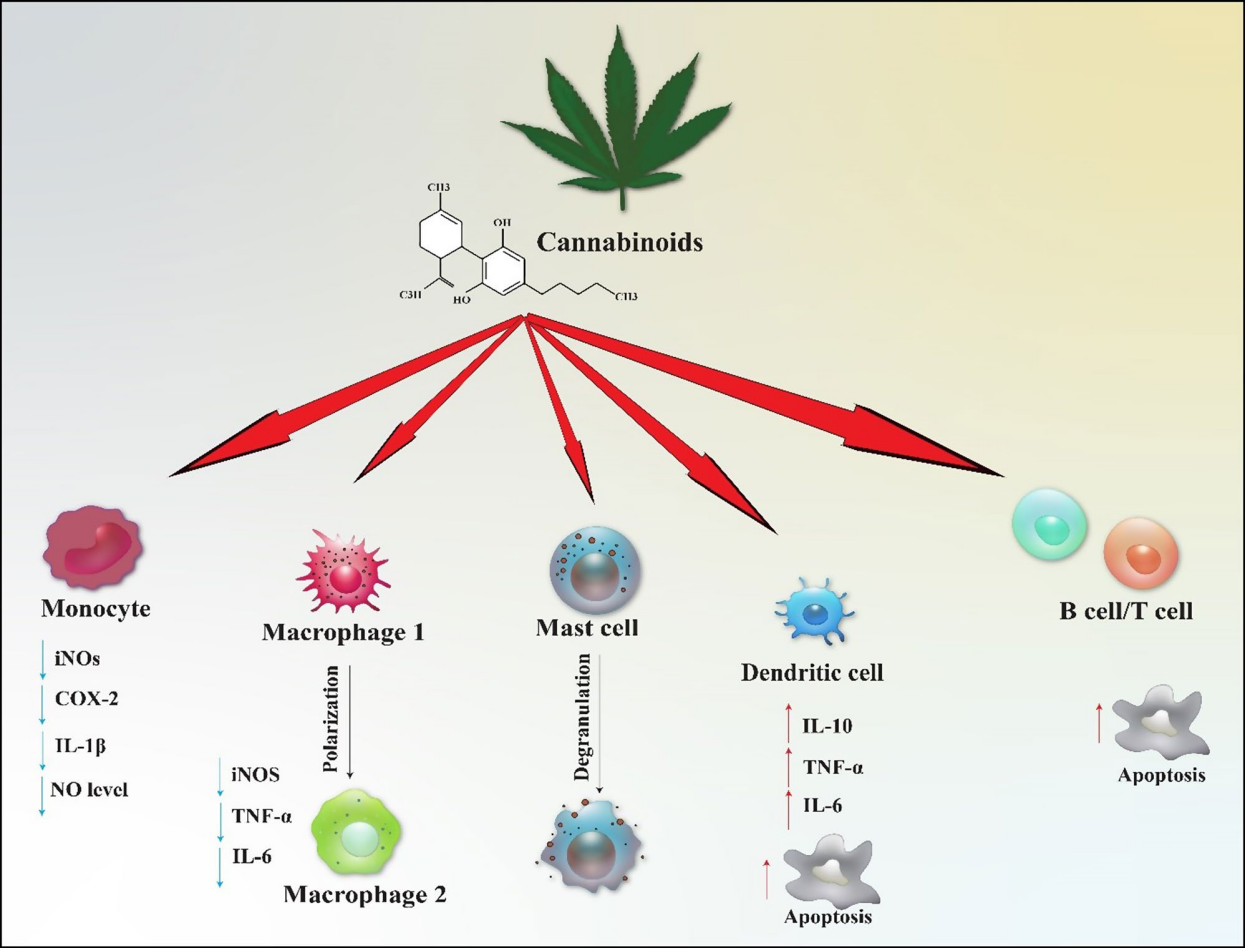
The impact of cannabinoids on immune cell function. The diverse impacts of endocannabinoids on immune cells are highlighted. In monocytes, CBD results in a reduction of IL-1β, COX-2, nitric oxide production, and iNOS activity. In macrophages, there is a noted shift from M1 to M2 polarization, accompanied by decreased levels of iNOS, IL-6, and TNF-α. Additionally, CBD enhances degranulation in mast cells. It also induces apoptosis in dendritic cells, which plays a role in immune regulation by increasing IL-10, TNF-α, and IL-6 levels. Furthermore, CBD promotes increased apoptosis in both B cells and T cells

The influence of phytocannabinoids on immune cells has been illustrated in Fig. 3. CBD is known to decrease NO levels in monocytes, which may reduce their inflammatory activity. It has been observed to decrease the expression of iNOS, COX-2, and IL-1β in monocytes. indicating a comprehensive anti-inflammatory effect. In macrophages, CBD has been shown to reduce the production of pro-inflammatory cytokines, thereby highlighting their potential role in alleviating inflammatory responses. However, it is important to note that CBD can also promote degranulation in mast cells, which may heighten inflammatory responses depending on the specific circumstances. It has been found to induce apoptosis in dendritic cells, which could have implications for antigen presentation and the maintenance of immune tolerance. Interestingly, CBD appears to elevate levels of both pro-inflammatory cytokines (such as IL-6 and TNF-α) and anti-inflammatory cytokines (like IL-10) in dendritic cells, suggesting a nuanced regulatory function. Furthermore, it has been shown to induce apoptosis in both B and T lymphocytes, potentially contributing to its immunosuppressive properties (Zorrilla et al. 2024). In a study by Chaoul et al. (2024), it was found that CBD can inhibit the activation of various immune cells in atopic dermatitis models. This leads to a decrease in pro-inflammatory cytokines and promotes cell death in B and T cells (Chaoul et al. 2024).

#### Immunoregulatory

CBD’s immunoregulatory properties extend beyond direct effects on iNOS. For instance, in an in vitro study investigating LPS-stimulated macrophages, CBD was shown to downregulate various pro-inflammatory mediators, including TNF-α and IL-6, while concurrently diminishing iNOS expression. The combination of CBD with other natural compounds, such as moringin, exhibited enhanced effects on inflammation markers and further supported the anti-inflammatory potential of CBD (Rajan et al. 2016).

Additionally, in vivo research indicated that CBD treatment was associated with reduced infiltration of T cells into the central nervous system following spinal cord injury. Specific immune markers linked to T cell activation, such as IL-23 and its receptor, were downregulated, suggesting that CBD influences the immune communication pathways vital for maintaining tissue homeostasis in the presence of injury or inflammation (Li et al. 2018). In addition, in *in vitro/in vivo* designed project reported that CBD increases levels of mitofusin 2, a mitochondrial fusion protein that plays a role in regulating inflammation and protecting against neurodegeneration. The upregulation of Mfn2 suggests an additional mechanism through which CBD exerts its anti-inflammatory effects, promoting an environment conducive to neuroprotection while limiting inflammatory responses mediated through microglial activation. Research also indicated that CBD treatment resulted in a reduction of TNF-α, IL-6, iNOS, COX-2, and ionized calcium-binding adaptor molecule-1 levels in microglia that had been challenged with LPS (Li et al. 2022). Recent research by Liu et al. (2024) in atherosclerosis models found that nano-CBD reprogrammed macrophage metabolism to reduce inflammation (Liu et al. 2024).

CBD’s interaction with various cellular signaling pathways further elucidates its immunomodulatory effects. For instance, the selective activation of PPARγ by CBD contributes to its anti-inflammatory actions, aligning with observed reductions in iNOS and pro-inflammatory markers in multiple studies (Filippis et al. 2011). This receptor’s involvement indicates that CBD facilitates an anti-inflammatory phenotype in immune cells, enhancing their ability to resolve inflammation.

CBD has diverse effects on immune cells, including reducing inflammation by decreasing NO and cytokine levels in monocytes and macrophages, promoting degranulation in mast cells, inducing apoptosis in dendritic cells and lymphocytes, and modulating cytokine production in dendritic cells. Recent studies suggest that CBD has immunoregulatory and immunosuppressive properties by downregulating pro-inflammatory mediators, reducing T cell infiltration, and promoting neuroprotection through pathways like PPARγ.

#### Modulatory effects on macrophage polarization and inflammatory response

CBD has a complex effect on immune response in chronic conditions like MS, reducing M1 markers while having a minimal impact on M2 markers. It also has dual effects on TNF-α and iNOS (Mujahid et al. 2025). In the exploration of CBD’s effects on macrophage polarization in the in vitro study, Frodella et al. investigated its impact on murine macrophages (RAW 264.7) under conditions of M1 (pro-inflammatory) and M2 (anti-inflammatory) polarization. In this recent investigation has revealed that CBD pretreatment demonstrates a complex modulatory effect on macrophage phenotypes and inflammatory responses. Specifically, CBD was found to decrease markers associated with pro-inflammatory M1 macrophages while having minimal impact on anti-inflammatory M2 macrophage markers. A particularly noteworthy finding was CBD’s dual effect on tumor TNF-α: while intracellular levels increased, the secretion of TNF-α was notably reduced. This phenomenon appears to be mediated through CBD’s influence on the cellular localization of TNF-α converting enzyme (TACE). Furthermore, CBD exhibits a complex interaction with iNOS activity, where it paradoxically promotes M1 characteristics through elevated iNOS levels while simultaneously demonstrating an overall anti-inflammatory effect through reduced TNF-α secretion. This intricate mechanism of action underscores the importance of carefully considering CBD’s therapeutic applications, particularly in treating chronic inflammatory conditions such as MS, where precise modulation of immune responses is critical. These findings contribute to our understanding of CBD’s immunomodulatory properties and suggest the need for further research to optimize its therapeutic potential in inflammatory diseases (Frodella et al. 2024). Cannabidiol represents a multifaceted tool in the modulation of the immune system, particularly through its effects on NOS and pro-inflammatory mediators. By downregulating iNOS expression and affecting the release of various cytokines, CBD offers significant anti-inflammatory and neuroprotective benefits. Continuing the exploration of CBD’s effects, Yeisley et al. in 2021 in the in vitro research examined how CBD alters the inflammatory protein landscape in macrophages activated by LPS. The study revealed that CBD treatment significantly reduced the expression of pro-inflammatory cytokines and proteins associated with oxidative stress, including eNOS. Notably, treatment with 25 µM CBD resulted in a reduction of eNOS expression, reinforcing the compound’s role in mitigating NO production (Yeisley et al. 2021). However, it is essential to note the differential roles of cannabinoids in modulating inflammation. While CBD exhibits a robust anti-inflammatory profile impacting NOS and NO pathways, other cannabinoids, such as CBG, may possess distinct mechanisms that do not primarily involve anti-inflammatory effects (Wen et al. 2023). Recent studies include Chen et al. (2024), where an injectable hydrogel with CBD inhibited M1 polarization in macrophages, reducing inflammation in tissue repair models (Wang et al. 2024).

Synthesizing these findings, CBD regulates macrophage polarization by reducing pro-inflammatory M1 phenotypes, affecting TNF-α secretion, and inhibiting NO production through eNOS/iNOS pathways. This demonstrates its complex immunomodulatory effects at different concentrations, making it a promising therapy for inflammatory conditions.

The comprehensive analysis of CBD’s effects on macrophage polarization and inflammatory responses reveals its sophisticated role as an immunomodulator. This section explained CBD’s ability to modulate both M1 and M2 macrophage phenotypes while exhibiting complex interactions with inflammatory mediators such as TNF-α and iNOS. CBD’s dual capacity to influence intracellular TNF-α levels while reducing its secretion, coupled with its effects on NO production through eNOS modulation, highlights its potential as a therapeutic agent. The differential response observed at various concentrations, provides valuable insights for dosing considerations in clinical applications.

### Effect of cannabidiol on NOS in metabolic syndrome

**Fig. 4:**
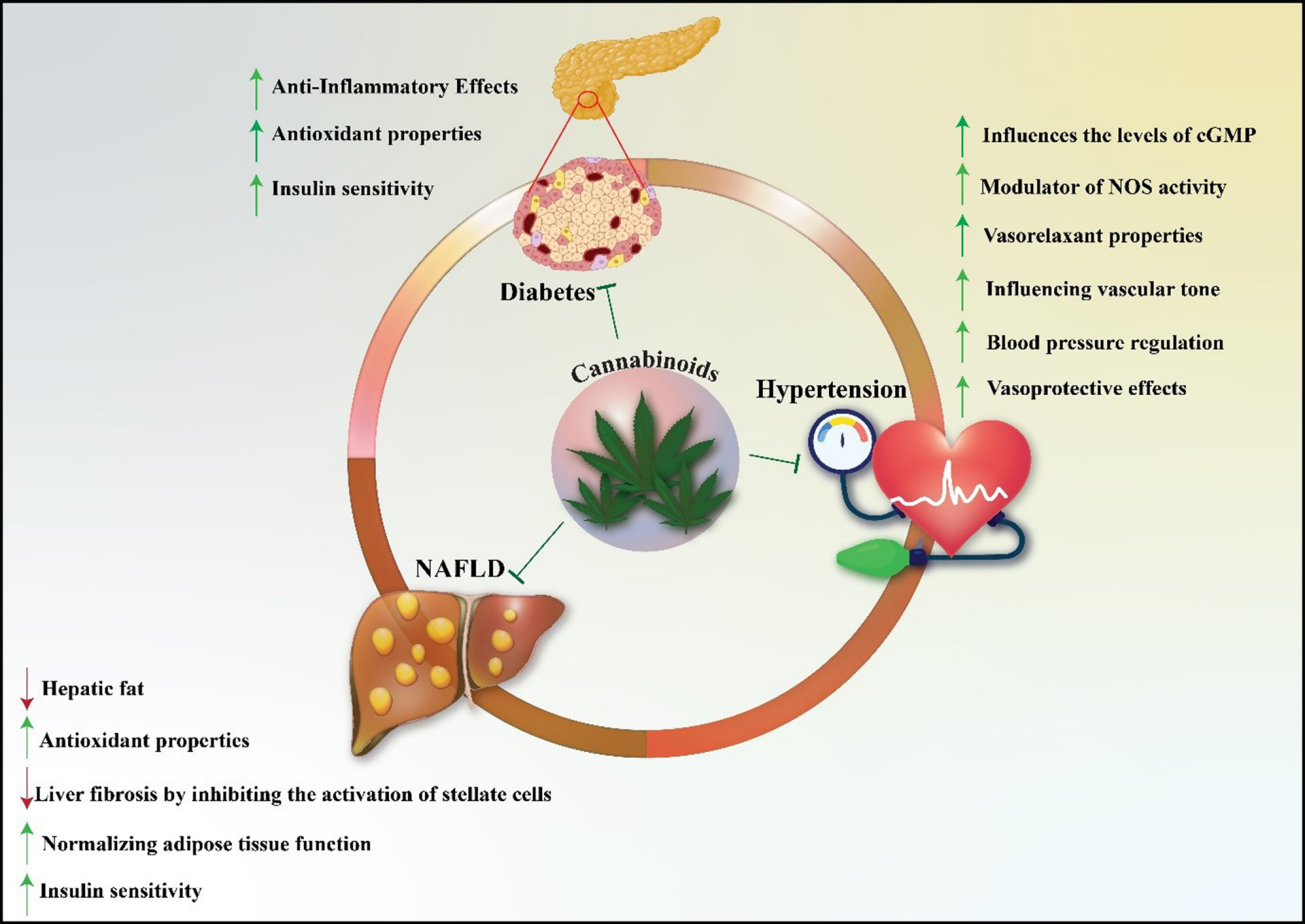
Therapeutic effects of CBD in metabolic disorders. CBD demonstrates multiple beneficial effects across three conditions: (Zhou et al. 2023) In diabetes, CBD enhances insulin sensitivity while providing antioxidant and anti-inflammatory properties; (Castillo et al. 2010) In hypertension, CBD exhibits vasoprotective effects through modulation of vascular tone, blood pressure regulation, vasorelaxation, NOS activity regulation, and cGMP level modification; (Gorelick et al. 2022) In NAFLD, CBD promotes metabolic health by normalizing adipose tissue function, increasing insulin sensitivity, and providing antioxidant effects, while reducing liver fibrosis through stellate cell inhibition and decreasing hepatic fat accumulation

Metabolic syndrome encompasses a group of interrelated conditions, including obesity, dyslipidemia, insulin resistance, and hypertension, often leading to an increased risk of type 2 diabetes and cardiovascular disease (Patil et al. 2020; Marzo and Silvestri 2019). A key player in the inflammatory processes associated with metabolic syndrome is NO, produced primarily by iNOS. The regulation of NO production is a crucial factor in both inflammatory and metabolic pathways, making CBD’s effects on iNOS particularly relevant (Gorelick et al. 2022; Matouk et al. 2017, 2018). The Fig. 4 demonstrates the multifaceted role of CBD in the management of metabolic disorders, specifically Non-Alcoholic Fatty Liver Disease (NAFLD), hypertension, and diabetes.

### NAFLD

NO, produced by the eNOS enzyme, plays a dual role in liver pathology; while it can have protective effects, excessive NO production often exacerbates inflammatory processes. For instance, studies on NAFLD models show that eNOS dysfunction contributes to endothelial impairment and insulin resistance, worsening steatosis (Sheldon et al. 2015). Similarly, elevated iNOS levels promote oxidative stress and inflammation in hepatic tissue, linking to NAFLD progression (Cunningham et al. 2021). Cannabinoids, particularly CBD, have shown promise in modulating these pathways, raising interest in their therapeutic potential for NAFLD (Wang et al. 2024). Moreover, a recent research investigates cannabinoid type 1 receptors (CB1Rs) in vascular function in hypercholesterolemic mice. Using a new double-knockout model (LDLR and CB1R), they found that CB1R deletion moderated damage to acetylcholine-induced vasorelaxation and attenuated high blood pressure caused by a high-fat diet. CB1R knockout also increased eNOS expression depressed by the high-fat diet (Vass et al. 2024).

Cannabinoid’s anti-inflammatory role extends to the liver, where elevated iNOS levels and NO production contribute to liver damage often associated with conditions like NAFLD. A recent study by Gorelick et al. investigated the distinct effects of CBD and THC on NAFLD progression using a high-fat-cholesterol diet animal model. The findings revealed that CBD administration significantly improved glucose tolerance and downregulated key inflammatory markers, specifically TNFα and iNOS. Although CBD treatment did not substantially reduce hepatic steatosis, its marked influence on inflammatory pathways suggests promising therapeutic potential. The study demonstrated that THC exhibited comparatively weaker effects on inflammatory gene expression, indicating that the therapeutic benefits of cannabinoids extend beyond their interactions with canonical cannabinoid receptors (Gorelick et al. 2022). In another study examining the effects of cannabis extracts on NAFLD, Assa-Glazer et al. in 2020 found that CBD-rich extracts increased inflammatory gene expression and altered gut microbiota profiles in mice fed a high-fat/cholesterol diet (HFCD). Notably, in contrast to numerous studies previously discussed where CBD decreased inflammation through reduced NO production and NOS activity, this study demonstrated that CBD administration was associated with elevated levels of inflammatory markers, including iNOS. Conversely, THC-rich extracts showed improvements in metabolic parameters, suggesting that the cannabinoid profile significantly influences the outcome in NAFLD (Assa-Glazer et al. 2020).

More recently, Yang et al. (2024) evaluated novel peripherally-restricted CB1 antagonists (PMG-505-010 and − 013) in a diet-induced obesity mouse model of NAFLD. These compounds improved metabolic profiles, reduced hepatic steatosis and inflammation (e.g., lowered cytokine levels), and inhibited fibrosis by decreasing extracellular matrix deposition, highlighting CB1 blockade as a mechanism to mitigate NAFLD without central nervous system side effects (Yang et al. 2024). Finally, a 2025 study by Degrave et al. analyzed five cannabis oils with varying CBD: THC ratios in a sucrose-rich diet rat model. THC-rich oils and balanced ratios (1:1, 2:1) effectively reduced hepatic steatosis, liver triglycerides, and inflammation (e.g., lowered AST/ALT), while also attenuating oxidative stress; CBD-rich oils showed antihypertensive effects but were less effective against steatosis (Degrave et al. 2025).

#### Mechanistic insight

In NAFLD, the balance of NO signaling plays a crucial role. eNOS-derived NO can be protective for the liver, but dysfunction of eNOS can lead to endothelial impairment, insulin resistance, and worsened fat accumulation in the liver (Sheldon et al. 2015). On the other hand, iNOS upregulation results in excessive NO production, leading to oxidative and inflammatory damage (Cunningham et al. 2021). Cannabinoid signaling influences these pathways in various ways. Activation of cannabinoid receptor type 1 (CB1) downregulates eNOS, contributing to vascular and metabolic damage (Vass et al. 2024). In contrast, blocking or deleting CB1 can restore eNOS expression and improve metabolic and fibrotic outcomes (Yang et al. 2024). CBD primarily exerts its effects through anti-inflammatory and NOS-suppressing actions, which can improve glucose tolerance and reduce inflammatory signaling (Gorelick et al. 2022). However, the overall effects of CBD may be influenced by factors such as the composition of the extract and individual host characteristics, including gut microbiota (Assa-Glazer et al. 2020). The specific composition of cannabinoids also impacts liver health outcomes. Peripheral CB1 antagonism and formulations containing THC or a balanced ratio of CBD to THC have shown greater effectiveness in reducing fat accumulation, inflammation, and oxidative stress compared to CBD-dominant oils in recent animal studies (Yang et al. 2024; Degrave et al. 2025).

In conclusion, current preclinical studies on CBD for NAFLD show conflicting results. Some studies suggest that CBD improves glucose tolerance and reduces inflammation by suppressing NOS, while others indicate pro-inflammatory effects from CBD-rich extracts. The outcomes seem to be influenced by the cannabinoid composition and targeting of CB1 receptors, highlighting the need for comparative mechanistic studies.

### Hypertension

Hypertension, a condition characterized by persistently elevated blood pressure, is a major risk factor for cardiovascular diseases. Its pathophysiology involves a complex interplay of genetic, behavioral, and environmental factors, often leading to endothelial dysfunction, increased vascular resistance, and ultimately, various cardiovascular complications (Berenyiova et al. 2022; Zhai et al. 2018; Chan and Chan 2014). NO plays a critical role in maintaining vascular homeostasis, and its synthesis is primarily mediated by NOS enzymes. In hypertension, the bioavailability of NO is often compromised, leading to endothelial dysfunction and increased vascular resistance (Berenyiova et al. 2022; Zhai et al. 2018; Chan and Chan 2014; Ryszkiewicz et al. 2025). Cannabidiol has emerged as a potential modulator of NOS activity, influencing vascular tone and blood pressure. This part synthesizes findings from recent studies to elucidate the effects of CBD on NOS enzymes in hypertensive settings.

Recently, Baranowska-Kuczko et al. in 2021 investigated the effects of chronic CBD treatment on endothelial function in hypertensive rats. This in vivo study demonstrated that CBD administration (10 mg/kg daily for 2 weeks) improved endothelium-dependent vasodilation in both deoxycorticosterone-induced hypertensive (DOCA-salt) rats and spontaneously hypertensive rats. The enhancement of vasorelaxation was significantly impaired by the eNOS inhibitor L-NAME, indicating a reliance on NO for the observed effects. Moreover, CBD treatment was associated with increased vascular expressions of cannabinoid receptors and elevated levels of endocannabinoids with vasorelaxant properties, such as anandamide and 2-arachidonoylglycerol. The study also highlighted the differential roles of NOS isoforms in various vascular beds. In aortic tissues, NO-mediated vasorelaxation was predominant, while mesenteric arteries showed variable dependence on NO, suggesting a complex interplay between CBD, NOS expression, and vascular reactivity. In aortic tissue, NO predominantly mediates relaxation, with L-NAME inhibition abolishing ACh-induced dilation. DOCA-salt rats exhibited slightly reduced NO-dependent relaxation compared to SHAM controls. Mesenteric G3 arteries from WKY rats and SHR demonstrated approximately 20% greater NO-mediated vasorelaxation than DOCA-salt rats. Vasodilator prostanoids maintained a minimal but consistent role in vascular tone regulation, particularly in normotensive conditions. These functional alterations corresponded with changes in eNOS expression patterns. DOCA-salt rats and SHR showed decreased aortic NOS3 expression without significant protein level changes. In mesenteric G3 arteries, NO pathway components varied by hypertension type, with primary hypertension showing enhanced NO-mediated relaxation through increased NOS3 and eNOS expression. DOCA-salt rats’ resistance arteries maintained stable eNOS levels. While NO-dependent relaxation showed clear hypertension-related modifications, COX-dependent responses remained unchanged in both functional and biochemical analyses (Baranowska-Kuczko et al. 2021). A recent project explores hemp (Cannabis sativa L.) leaf oil (HLO)’s cardiovascular benefits in hypertensive rats. HLO prevented blood pressure increase, improved heart function, and reduced cardiac hypertrophy. It also improved vascular function by reducing vasoconstriction and increasing vasorelaxation. HLO inhibited the renin-angiotensin system (RAS), decreased oxidative stress, and enhanced antioxidant status. These results suggest HLO protects against cardiovascular dysfunction by suppressing RAS and oxidative stress through the Ang II/AT1 receptor/NOX2 pathway (Khamseekaew et al. 2025). In a rat metabolic syndrome model, a CBD-dominant cannabis oil reduced hypertension and dyslipidemia. The authors noted these oils likely improve vascular tone via terpene-enhanced NO release (Degrave et al. 2025). Though not a pure CBD study, it supports CBD’s role in NO-mediated vasodilation.

In vivo research by Matouk et al. in 2017 demonstrated that chronic activation of GPR18 with abnormal cannabidiol (abn-CBD) led to hypotension and improved cardiac function in rats. The mechanism involved an increase in eNOS expression and NO production, contributing to the observed reductions in blood pressure. Enhanced levels of cGMP were noted, supporting the involvement of NO as a signaling molecule in mediating vasodilation (Matouk et al. 2017).

Moreover, Penumarti and Abdel-Rahman further elucidated the role of GPR18 in modulating NOS activity within the rostral ventrolateral medulla (RVLM), a critical area for blood pressure regulation. Activation of GPR18 increased nNOS phosphorylation and NO levels, leading to reduced oxidative stress and blood pressure. This underscores the potential for GPR18 as a novel target for hypertension therapy (Penumarti and Abdel-Rahman 2014). Moreover, in 2018 an in vivo research by Matouk et al. investigated the effects of abnormal cannabidiol (abn-CBD) on cardiovascular health in diabetic rats (Matouk et al. 2018). Although abn-CBD did not affect glycemic control, it significantly improved cardiac function and reduced oxidative stress. The mechanism involved the restoration of NO levels through enhanced eNOS signaling, which was associated with the activation of GPR18. Importantly, the favorable effects of abn-CBD were abrogated by GPR18 antagonism, highlighting the significance of GPR18 in mediating the cardiovascular benefits of cannabinoids (Matouk et al. 2018).

#### Mechanistic insight

In hypertension, dysfunctional eNOS and excess oxidative inactivation reduce NO bioavailability, leading to endothelial dysfunction and increased vascular resistance (Berenyiova et al. 2022; Zhai et al. 2018; Chan and Chan 2014; Ryszkiewicz et al. 2025). Cannabinoids like CBD and abn-CBD restore NO signaling by upregulating eNOS, increasing cGMP levels, and promoting vasorelaxation through elevated endocannabinoid levels and GPR18 activation. These effects are NOS-dependent and can be reversed by L-NAME or GPR18 antagonism (Baranowska-Kuczko et al. 2021; Matouk et al. 2017, 2018; Penumarti and Abdel-Rahman 2014). Additionally, cannabinoids suppress the Ang II/AT1/NOX2 axis to reduce oxidative stress and RAS activity (Khamseekaew et al. 2025), while terpenes in complex oils facilitate NO release (Degrave et al. 2025). This results in improved endothelial-dependent vasodilation and blood pressure reduction, with effects varying depending on the vascular bed and NOS isoform.

Cannabidiol demonstrates promising vasoprotective effects in hypertensive models, primarily through modulation of NOS enzymes. CBD enhances endothelial function and NO bioavailability, contributing to improved vascular reactivity and blood pressure regulation. Additionally, the activation of GPR18 may further amplify these effects through increased nNOS activity and reduced oxidative stress.

### Diabetes

Diabetes is a multifaceted disease characterized by chronic hyperglycemia, leading to various complications, including cardiovascular disorders and cognitive impairments. The dysregulation of NO production due to altered NOS activity is a critical factor in the pathogenesis of these complications.

Recent review report that CBD suppresses diabetes-driven iNOS/NO overproduction. Lee et al. (2024) highlight studies where CBD normalized cardiac function in diabetic rats by inhibiting iNOS and downstream oxidative pathways (Lee et al. 2024). Previously an in vivo research by Rajesh et al. in 2007 demonstrated that CBD significantly attenuates the inflammatory response induced by high glucose (HG) in human coronary artery endothelial cells (HCAECs) (Rajesh et al. 2007). HG exposure led to increased mitochondrial superoxide generation, activation of the NF-κB pathway, and an upregulation of iNOS and adhesion molecules (ICAM-1 and VCAM-1). These changes were associated with disrupted endothelial barrier function and enhanced monocyte adhesion. However, CBD pretreatment effectively reversed these effects, suggesting that it may preserve endothelial integrity and function by modulating iNOS activity and reducing oxidative stress (Rajesh et al. 2007).

In other in vivo study, in middle-aged diabetic rats exposed to chronic cerebral hypoperfusion, CBD treatment improved cognitive performance and reduced neuroinflammation markers, including iNOS in the hippocampus (Santiago et al. 2019). While the study indicated that CBD did not significantly affect neuroplasticity markers, the reduction in iNOS suggests a critical role for CBD in mitigating neuroinflammatory processes associated with diabetes (Santiago et al. 2019). However, based on the previous studies several mechanisms for the beneficial effects of CBD through NOS regulation have been porposed in diabetes management. These mechanisms include: (1) Reduction of Oxidative Stress: Both CBD and abn-CBD have been shown to alleviate oxidative stress in diabetic models, which is crucial for maintaining NO bioavailability and preventing endothelial dysfunction. (2) Modulation of Inflammatory Pathways: CBD’s ability to inhibit inflammatory mediators, including iNOS, suggests that it can help restore a balance in NO production, favoring protective over harmful pathways. (3) Activation of Signaling Cascades: The activation of the PI3K/Akt/eNOS pathway by cannabinoids enhances NO production, contributing to improved vascular function and cardioprotection (Matouk et al. 2018; Rajesh et al. 2007; Santiago et al. 2019).

#### Mechanistic insight

CBD in diabetes restores NO homeostasis by suppressing pathological iNOS-derived NO and supporting protective eNOS signaling. This is achieved through the inhibition of NF-κB and adhesion molecule upregulation, reduction of mitochondrial ROS, preservation of endothelial barrier integrity, and activation of PI3K/Akt→eNOS pathways (Matouk et al. 2018; Lee et al. 2024; Rajesh et al. 2007; Santiago et al. 2019). CBD also reduces neuroinflammation and oxidative stress in diabetic brain models, leading to improved cognition and reduced neuroinflammatory damage (Santiago et al. 2019).

In conclusion, CBD shows therapeutic promise for diabetes complications by modulating NO pathways. Studies demonstrate its anti-inflammatory and antioxidant effects, ability to regulate NOS activity, and protection of endothelial function against high glucose-induced damage. CBD helps restore NO balance through multiple mechanisms, suggesting potential as a diabetes treatment, though more clinical research is needed.

### CBD formulations or delivery systems

The formulation and route of administration significantly impact the pharmacokinetics, tissue distribution, and pharmacodynamic profile of CBD. Due to CBD’s high lipophilicity and extensive first-pass metabolism, various delivery platforms have been developed to enhance solubility, protect CBD from degradation, facilitate lymphatic uptake, and modulate systemic versus local exposure (Stella et al. 2021). These platforms include oil solutions, self-nanoemulsifying drug delivery systems (SNEDDS)/nanoemulsions, solid-lipid and nanostructured lipid carriers (SLNs/NLCs), polymeric nanoparticles (e.g., PLGA, PCL), cyclodextrin inclusion complexes, nanocrystals, and topical ethosomal/ethosomal-like vesicles (Stella et al. 2021).

Human data demonstrate that SNEDDS/nanoemulsion approaches can significantly increase relative bioavailability compared to conventional oil drops, leading to faster absorption and higher peak concentrations of CBD and its active metabolite 7-OH-CBD (Hermush et al. 2025; Assadpour et al. 2023). Sustained-release oral matrices and transdermal or topical nanocarriers result in lower peak concentrations and prolonged exposure, which may be beneficial for chronic conditions requiring steady exposure and reduced peak-related adverse events (Stella et al. 2021; Banerjee et al. 2025).

Formulation influences metabolite patterns and first-pass biotransformation, impacting efficacy and safety. Matching the delivery strategy to the disease and mechanism of action is crucial, with intranasal/parenteral or highly bioavailable oral nanoformulations suitable for rapid CNS indications, while transdermal/colloidal topical systems are ideal for localized inflammation. Future comparative studies should include formulation details, particle characteristics, pharmacokinetics, and tolerability to facilitate meaningful cross-study interpretation (Stella et al. 2021; Banerjee et al. 2025).

### Overview of CBD safety concerns

While CBD shows therapeutic potential, clinical trials and product labels have reported common adverse effects such as gastrointestinal symptoms, fatigue/somnolence, and appetite/weight changes, which are usually dose-related and can improve with dose adjustment (Zhou et al. 2023; Kodali et al. 2024). More concerning are findings from systematic reviews and meta-analyses indicating dose-dependent hepatotoxicity and an increased risk of clinically significant liver enzyme elevations, especially when CBD is taken with drugs like valproate that share metabolic pathways. Therefore, monitoring liver function and reviewing concomitant medications are crucial (Zhou et al. 2023; Kodali et al. 2024; Lo et al. 2023). Recent randomized data have shown liver enzyme elevations in healthy adults using typical CBD doses, highlighting that the risk is not limited to epilepsy trials or high doses (Florian et al. 2025). Preclinical studies also suggest potential toxicity at high doses in animal models, emphasizing the need for caution when extrapolating safety from animals to humans (Lo et al. 2023).

Furthermore, research has revealed that primary CBD metabolites, particularly 7-OH-CBD and 7-COOH-CBD, can impact neural stem cells and potentially contribute to cytotoxicity or enzyme inhibition, leading to prolonged exposure (Latham et al. 2025).

### Epigenetic regulatory mechanisms

While epigenetic studies on CBD are still emerging, it is important to consider its potential impact on inflammation, oxidative stress, and nitric oxide signaling. CBD has shown to modulate redox-sensitive transcription factors like Nrf2 and NF-κB, which are regulated by epigenetic mechanisms such as histone marks and DNA methylation in disease conditions (Atalay Ekiner et al. 2022). NO pathways, including inducible and endothelial NOS, are also linked to epigenetic changes like histone acetylation, DNA methylation, and microRNA expression that affect gene expression and cellular functions (Socco et al. 2017).

Although the direct epigenetic effects of CBD on NOS or Nrf2 signaling have not been fully elucidated, the overlap between CBD’s redox and anti-inflammatory properties with epigenetically controlled pathways suggests a potential mechanistic connection. Future research should investigate whether CBD influences epigenetic regulators like histone-modifying enzymes, DNA methyltransferases, or microRNA networks in NOS and oxidative/inflammatory pathways. Exploring these epigenetic mechanisms could lead to new insights into CBD’s regulatory roles and deepen our understanding of its effects.

### Limitations

This review summarizes preclinical and limited clinical data on CBD’s effects on NOSHowever, there are critical limitations that challenge the translation of these findings to clinical applications. Preclinical studies often use high CBD concentrations that exceed physiologically achievable levels in humans, raising doubts about clinical relevance. Clinical evidence on CBD’s effects on NOS isoforms and NO signaling is sparse and heterogeneous, limiting conclusions for human use. Mechanistic assumptions about CBD-NOS interactions may oversimplify the effects, which could be influenced by other pathways. CBD’s impact on NOS varies depending on cell type, tissue environment, and disease state, sometimes promoting pro-inflammatory signals. Publication bias and variability in study formulations make cross-study comparisons challenging. Understanding how in vitro findings relate to human exposures is crucial due to metabolic and tissue distribution differences between laboratory models and clinical settings.

### Future directions

To advance the clinical utility of CBD, future research should focus on establishing pharmacokinetically relevant dose-exposure mapping and standardizing CBD preparations. Well-designed clinical trials are needed to assess CBD’s modulation of specific NOS isoforms in patients with cardiovascular and neuroinflammatory conditions. Biomarkers should be incorporated to establish mechanistic correlations between CBD exposure and biological effects. Research should identify the most promising NOS isoforms as therapeutic targets and explore dose optimization and combination therapies. Addressing publication bias and developing translational models will provide a more balanced assessment of CBD’s efficacy. Long-term safety monitoring in NOS-targeted trials is crucial. These efforts will bridge the gap between preclinical mechanisms and therapeutic applications.

## Conclusion and perspective

In summary, the ongoing exploration of CBD’s interaction with NOS and its broader implications for human health underscores the need for rigorous scientific inquiry. As we continue to unravel its potential, the integration of cannabinoid-based therapies into mainstream medical practice could revolutionize approaches to treating chronic diseases characterized by inflammation and oxidative stress. Future research initiatives must aim to refine our understanding and expand the clinical applications of CBD, ultimately contributing to more effective and comprehensive healthcare solutions. This narrative review elucidates the modulatory effects of CBD on NOS and its therapeutic implications for managing inflammation and oxidative stress-related disorders. CBD selectively influences the expression and activity of iNOS and eNOS, thereby regulating NO production, which is essential for cardiovascular health, neuroprotection, and immune responses. The evidence presented highlights CBD’s potential to counteract oxidative stress and inflammation across various pathophysiological contexts, including cardiovascular diseases, neurological disorders, cancer, and metabolic syndromes. Additionally, the dysregulation of NOS isoforms is shown to contribute to the pathogenesis of these diseases, and CBD’s modulation of these pathways may alleviate endothelial dysfunction and improve vascular tone. Furthermore, this article emphasizes CBD’s diverse roles, including its antioxidant, anti-inflammatory, vasorelaxation, immune suppression, antinociceptive, neuroprotective, and cardioprotective effects. These findings suggest that CBD may serve as a promising cannabinoid-based therapeutic agent in treating chronic diseases associated with inflammation and oxidative stress. Overall, the review underscores the need for further research to explore the clinical applications of CBD and its mechanisms in various health scenarios, paving the way for evidence-based treatments that harness the therapeutic potential of cannabinoids.

## Data Availability

No datasets were generated or analysed during the current study.
